# Patient-derived organoids based on targeted biopsy of primary prostate cancer: development, identification, and drug screening

**DOI:** 10.1080/07853890.2025.2602324

**Published:** 2025-12-16

**Authors:** Jiajun Qian, Shan Peng, Jie Gao, Yao Fu, Haifeng Huang, Cheng Wu, Weidong Gan, Xuefeng Qiu, Hongqian Guo

**Affiliations:** ^a^ Department of Urology, Affiliated Drum Tower Hospital, Medical School of Nanjing University, Nanjing, China; ^b^ Institute of Urology, Nanjing University, Nanjing, China; ^c^ Department of Pathology, Affiliated Drum Tower Hospital, Medical School of Nanjing University, Nanjing, China; ^d^ Department of the Comprehensive Cancer Centre, Affiliated Drum Tower Hospital, Medical School of Nanjing University, Nanjing, China

**Keywords:** organoids, prostate cancer, primary, targeted biopsy, precise medicine

## Abstract

**Objectives:**

To develop patient-derived organoids (PDOs) using biopsied tissue of primary prostate cancer (PCa).

**Methods:**

Fresh tumour tissues of PCa were obtained from patients who underwent targeted biopsy in our centre for the culture of PDOs. Hematoxylin-Eosin (H & E) and immunohistochemical staining were used to determine the histology of the cultured PDOs, using the parental tumours as the reference. Whole exome sequencing (WES) was conducted to verify the genetic conservation of PDOs, using the parental tumours as the reference. Drug screening was carried out to test the feasibility of PDOs as preclinical models.

**Results:**

H & E and immunohistochemical staining indicated similar pathologic features between our developed PDOs and the parental tumours. WES also demonstrated similar somatic mutations, base substitutions and copy number variations between PDOs and the parental tumours. Drug screening results showed heterogeneity of the cultured PDOs to enzalutamide, docetaxel, and olaparib.

**Conclusion:**

PDOs can be successfully developed based on targeted biopsy of primary PCa, which may be an optimal preclinical model to predict treatment response in the era of precision medicine.

## 1. Introduction

The optimal treatment options or combination strategies for the treatment of prostate cancer (PCa) in the era of precision medicine mainly rely on understanding the specific tumour characteristics and biology, especially the molecular signatures [1,2]. Apparently, the reliable and repeatable approaches to obtain and capture tumour tissues for molecular profiling are the key basis of the precise treatment of PCa. Immortalized cell lines or patient-derived xenograft (PDX) are commonly used models to identify the molecular signatures and test treatment responses. Though cell lines cultured *in vitro* are available and simple, they are apparently far from being qualified to represent the heterogeneity between individuals. PDX, implanting patient-derived tumour tissue into an immunodeficient mouse, mimics the individual’s tumour characteristics. However, the establishment of PDX is inefficient and time-consuming, which is not suitable for large-scale applications. PDXs are still limited to display the complexity observed in primary tissues, despite being good models of the human disease [3]. Most importantly, the immune environment, which plays a key role in the tumour progression and drug resistance [4,5], is totally different between PDX models and clinical situations [6].

In the past decade, three-dimensional models of tumour tissue from patients, called patient-derived organoids (PDOs), which are more similar to the genetic lineage of tumours *in vivo* and the characteristics of naturally occurring tumours, such as tissue structure, cell phenotype, heterogeneity and drug response, have been developed [7]. In the field of PCa, PDOs have been successfully established by using metastatic tissues (e.g. bone, lymph node, pleural effusion, soft tissue, circulating tumour cell), radical prostatectomy specimens, and also benign prostate tissues [8–13]. However, the large-scale application of RP specimen- or metastases-derived PDOs is challenging due to the limited access to tumour tissues. In contrast, prostate biopsy is a more practical procedure to obtain primary PCa tissues, especially in the magnetic resonance imaging (MRI) era. Currently, targeted biopsy in combination with systematic biopsy is recommended for patients with positive lesions on MRI [14]. However, the feasibility and application of biopsy-tissue-derived PDOs have not been investigated yet.

Therefore, we developed PDOs using targeted biopsy-based tumour tissue. Histopathology and molecular sequencing were used to identify our constructed PDOs. Furthermore, PDOs were exposed to common drugs for the treatment of PCa to demonstrate individual treatment responses.

## 2. Materials and methods

### 2.1. Sample collection

**Figure 1.**
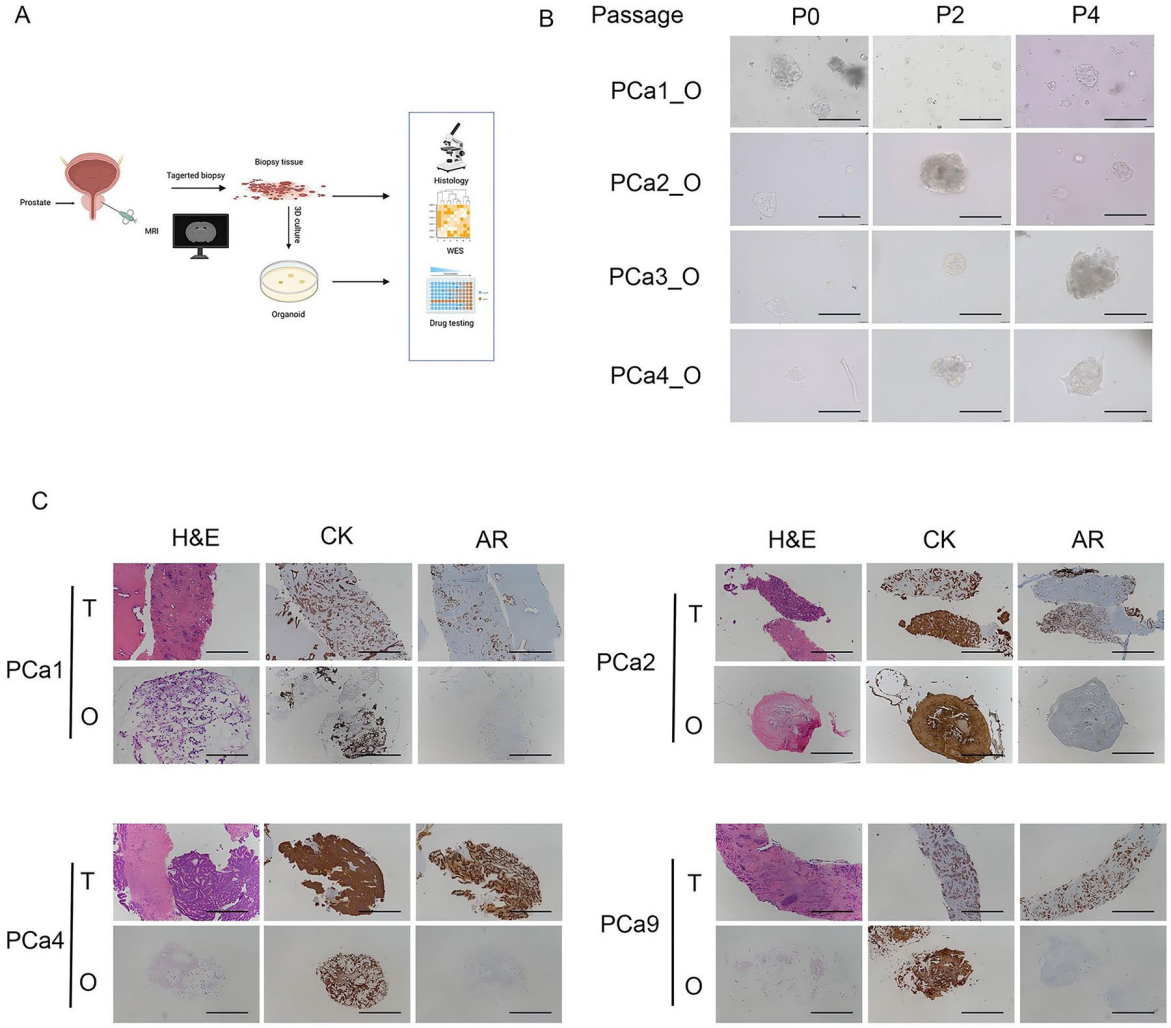
Histopathological characterization of prostate cancer PDOs. (A) Overview of experimental design. (B) Staining smear of prostate cancer PDOs. Scale bars, 200 µm. (C) H&E staining and immunohistochemistry staining of CK and AR on prostate cancer PDOs and corresponding parental tumours. Scale bars, 100 µm.

Fresh PCa biopsy specimens were obtained from patients who signed an informed consent form, with PSA > 10 ng/ml and positive lesions (PI-RADS score of 4 or 5) on MRI in Nanjing Drum Tower Hospital from July 2022 to June 2023. Briefly, after a standard 12-core systematic biopsy and MRI/ultrasound fusion-guided targeted biopsy, which was performed according to the previously described protocol [15], two additional specimens of targeted biopsy were obtained from the largest lesion for further investigations (Figure 1A). The study has been approved by the Ethics Committee of Nanjing Drum Tower Hospital (2022-631).

### 2.2. Organoid culture

The sample tissues were transferred to the 50 ml centrifuge tube and cleaned 3–5 times with 20–30 ml sterile saline containing 1% diamantine and 50 μg/ml gentamicin. The tissues were cut into small pieces of 1 mm by sterile scissors on the ice box. The small pieces of tissues were collected in a 15 ml centrifuge tube with HBSS, which were centrifuged at 1200 rpm for 3 min. After discarding the supernatant, 3 ml of digestive liquid was added to the precipitate and digested in a shaking bed at 37 °C for 0.5–3 h. 5 ml HBSS was added to terminate digestion after the first digestion. The tissues were centrifuged at 1200 rpm for 3 min, and the supernatant was discarded. Then, 2 ml preheated digestive fluid II was added to the precipitation and digestion was continued for 5–10 min in a shaking bed at 37 °C. After digestion was completed, 5 ml HBSS was added to terminate digestion and centrifuged at 1200 rpm for 3 min for discarding the supernatant. 5 ml HBSS was added for re-suspension precipitation, and the filtrate was collected using a 100 μm cell screen, which was centrifuged at 1200 rpm for 3 min to harvest cell precipitation. The cell precipitates were suspended by adding 2 ml erythrocyte lysate, leaving at room temperature for 2–5 min. Then, 4 ml HBSS solution was added to dilute and terminate dissociation. The cell precipitates were harvested by centrifuging at 1200 rpm for 3 min. Cell suspensions of 10^4 ∼ 10^5 cells/30ul were prepared by using a small amount of culture medium to re-suspend and dilute cell precipitates. An appropriate volume (no more than 200 μL) of collagen P (pre-melted at 4 °C) and cell suspension (no more than 200 μL) was mixed thoroughly according to the ratio of 1:1.5. The glue mixed with cells was dropped into a 60 mm culture dish by pipette, about 50 μL per drop. (To prevent collagen P from solidifying, it should be placed in the ice box.) The inoculated culture dish was placed in a CO_2_ incubator at 37 °C for 2 min. After shaking the glue without obvious flow, the culture dish was carefully inverted until it was fully solidified (about 30 min). 4 ml culture solution was added and cultured in an incubator at 37 °C and 5% CO_2_ concentration. The culture medium was replaced every 3–5 days so that PDOs could be obtained by 4–10 days in general.

### 2.3. Immunohistochemistry

More than 10^^^5 cells were collected for organoid verification. The organoid precipitation was collected, cleaned with PBS, and fixed with 4% paraformaldehyde, then embedded with 20–50 μL 2% agarose solution. Briefly, the organoid tissue was dehydrated, transparentized, paraffin-embedded, then sliced for subsequent Hematoxylin-Eosin (H&E) staining, immunohistochemical staining. Primary antibodies to AR, CK (Cat#ZA-0554, ZA-0069, respectively, ZSGB-BIO, China) were used for IHC on formalin-fixed paraffin-embedded tissue slides from the prostate cancer organoid. All the pathological sections are reviewed and confirmed by 3 experienced pathologists.

### 2.4. Whole-exome sequencing and analysis

Using the QIAGEN DNeasy Blood and Tissue Kit (QIAGEN) or QIAamp DNA FFPE Tissue (QIAGEND) to extract total DNA from cancer tissue, adjacent non-cancer tissue, and cultured organoids. Subsequently, fragmentized DNA is subjected to sequencing library preparation using the Covaris M220 Focused-ultrasonicator (Covaris). Following the manufacturer’s recommended protocol, exon capture is performed using the SureSelect Human All Exon V6 kit (Agilent Technologies). Sequencing is carried out on the Illumina Novaseq 6000 (LC BioTech Corporation) using a 150-bp paired-end sequencing mode. Before alignment, the low-quality reads (including the sequencing adapters and nucleotides with a q quality score lower than 20) were removed by fastp [16]. In the alignment step, the Burrows-Wheeler Aligner [17] was employed to align the reads to the reference genome hg19. As the initial post-alignment processing step, the Picard tool (http://broadinstitute.github.io/picard/) was used to identify and mark duplicate reads from the BAM file. Subsequently, in the second step, local read realignment was performed to correct potential alignment errors around indels. Base quality score recalibration was then conducted before variant calling to reduce systematic biases. Somatic SNVs and InDels were jointly called by Mutect2 [18] and Strelka [19], with only variants passing through two quality filters entering subsequent analysis. The Variant Effect Predictor [20] was utilized to incorporate biological information into the variant set. Control FreeC [21] detected copy number changes. The GC content of the sequences was used to normalize read distribution, and a normalized distribution of aligned reads in sliding windows was used to calculate copy number differences between tumour and normal samples.

### 2.5. Organoid drug screening

The treatment of anticancer drugs and sensitivity tests followed a previously described procedure [22], with some modifications. Briefly, organoids were harvested and dissociated using TrypLe (Gibco, USA). Dissociated organoids were combined with Matrigel (Corning) in OCOM (Accuroid, China) (1:1, v/v) and added to 384-well black plates with clear bottoms (Corning, USA). After gelation, 50 µL organoid culture media was added to each well. Organoids were cultured for 48 h. The compounds were then diluted in series (Olaparib: 4-0.0039 μM, docetaxel: 10-0.0032 μM, enzalutamide: 10-0.0032 μM) using the Robotics software. Cell viability was assessed using 3D CellTiter-Glo (Promega, USA) after 96 h of incubation. GraphPad Prism 8.0 (GraphPad Software, USA) was used to determine the IC50 values.

## 3. Results

### 3.1. Establishment of PDOs based on targeted biopsy of primary prostate cancer

We obtained fresh biopsy tissues from 13 patients with a significantly high level of PSA and suspicious lesions on MRI. Among them, 12 patients were finally diagnosed with prostate adenocarcinoma. No tumour was detected in one patient (#6 patient), who was excluded from further investigations. The detailed information of the included 13 patients was summarized in Supplementary Table 1. PDOs were successfully cultured from all 12 samples. Among them, 9 cases with good proliferation were used for the following pathologic identification, whole exon sequencing, and drug screening, while the remaining 3 cases with limited proliferation were used for identification only.

As shown in Figure 1B, PDOs were quickly formed and maintained the same morphological appearance in culture over passages, despite slower growth and smaller size (P0–P4). H&E staining showed densely arranged atypical small glands and cells, suggesting the successful culture of PDOs (Figure 1C). Immunohistochemistry of PDOs and their corresponding parental biopsy specimen showed a high degree of consistency in the marker expression of CK and androgen receptor (AR) (Figure 1C).

### 3.2. PDOs recapitulate the mutational spectrum of the corresponding parental PCa

**Figure 2.**
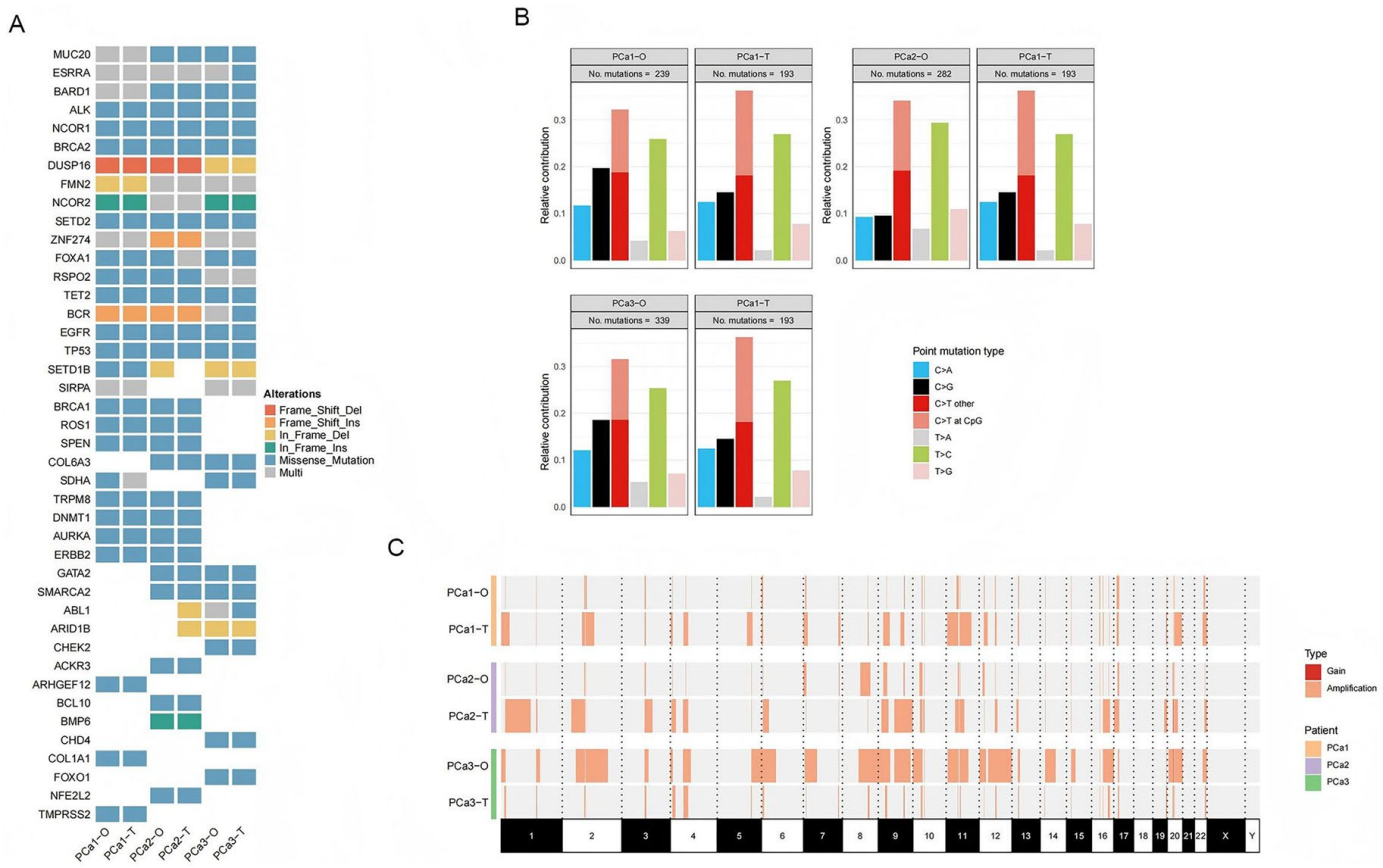
Genetic alterations of patient-derived prostate cancer organoid. (A) Somatic genomic mutation of prostate cancer PDOs and the corresponding parental tumours. (B) Point mutation types of prostate cancer PDOs and the corresponding parental tumours. (C) Genome-wide gene copy number variations (CNVs) of prostate cancer PDOs and the corresponding parental tumours.

Whole-exome sequencing (WES) was performed to investigate whether PDOs had similar genetic mutations to their parental tumours. The results were analyzed by comparing samples of PDOs and their parental tumours against matched normal blood samples. To demonstrate the genetic conservation between PDOs and their parental tumours, we analyzed related frame shift deletion, missense and nonsense mutation, somatic base substitutions and copy number variations (CNVs). Most somatic mutations in the corresponding parental tumour tissue were preserved in the PDOs (Figure 2A). The most common mutations are missense mutations, including TP53, EGFR, and BRCA2, present in all PDOs and parental tumours (Figure 2A). Other prostate cancer-associated somatic mutations were consistent in some PDOs and parental tumours, such as BRCA1, ALK, STED2, TET2, TRPM8, AURKA, ERBB2, GATA2. In addition, missense mutations of TMPRSS2 were only found in patient #1, while NFE2L2 was only found in patient #2 and FOXO1 was only found in patient #3. However, FOXA1 mutation was detected in all samples except patient #2.

Similar somatic base substitutions between PDOs and corresponding parental tumours were found in the three samples of PDOs and their corresponding parental tumours (Figure 2B). Also, similar results were found in terms of DNA gain and amplification patterns by CNV analysis (Figure 2C).

### 3.3. Drug response of prostate cancer organoids

**Figure 3.**
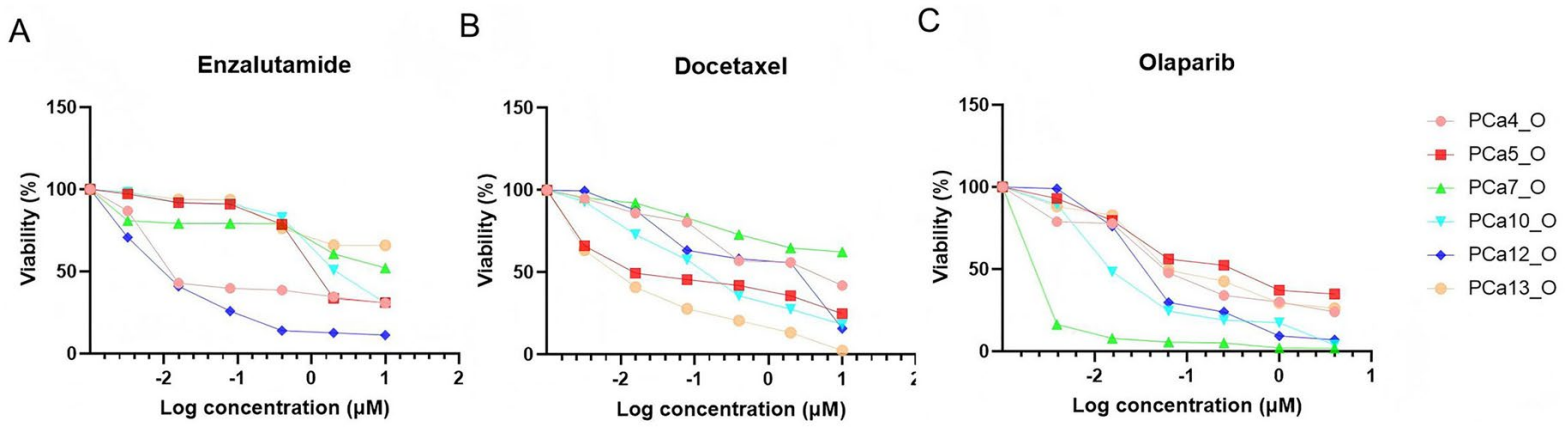
Drug screening of patient-derived prostate cancer PDOs. Dose-response curves of prostate cancer PDOs treated with (A) enzalutamide, (B) docetaxel and (C) olaparib.

Figure 3 shows the drug response of our cultured PDOs to common drugs for the treatment of PCa. Generally, PDOs showed good response to enzalutamide (Figure 3A), docetaxel (Figure 3B), and olaparib (Figure 3C). However, the response of each PDOs showed significant heterogeneity. For instance, PDOs of #7 patient exhibited a poorer response to both enzalutamide and docetaxel, but good response to olaparib, compared to the other PDOs. PDOs of #13 patient showed good response to docetaxel but poor response to enzalutamide.

## 4. Discussion

In the present study, we successfully established PDOs using the biopsied primary PCa tissues. Further analyses confirmed that our developed PDOs summarized similar histologic and molecular features with the parental tumours. In addition, drug sensitivity assay results suggested that targeted biopsy-derived PDOs could serve as an optimal model for precision medicine. In addition, our developed PDOs could be used to elucidate the mechanism of drug resistance and screen for gene mutations that lead to the emergence and development of disease. To the best of our knowledge, this is the first study to develop the PDOs of primary PCa using targeted biopsy-based tumour tissues.

In recent years, PCa organoids development has been reported by using tumour tissue from RP specimens [12] and also metastases [10]. However, the main limitation of these models for precision medicine is the limited access to tumour tissues. As we know, only a small part of patients would receive RP as the curative treatment. In addition, it is impossible to obtain tumour tissue from RP specimen. Compared to RP specimen, the biopsy specimen is an optimal alternative to obtain and capture tumour tissues since it is more reliable and repeatable. In the era of MRI, primary PCa lesions are “visual”, making it possible to biopsy tumour tissue accurately. In fact, the consistency of pathology between PDOs and parental tumour biopsy was 100% (12/12), suggesting the feasibility of targeted biopsy-derived PDOs. Liquid biopsy utilizes non-invasive sampling to acquire tumour information, aiding in cancer treatment and potentially replacing traditional biopsy techniques [23,24]. However, it would be difficult to obtain sufficient tumour cells from the blood of PCa patients to develop PDOs. Till now, very few studies have reported the application of circulating tumour cells obtained from PCa patients to develop PDOs [9].

The other issue regarding the culture of PDOs is the tissue volume. Unlike RP or salvage lymph node dissection, biopsy-derived tissue volume is limited. In the present study, two additional specimens of targeted biopsy were obtained for the culture of PDOs. Of the 13 included patients, PDOs were successfully cultured in all cases with PCa, with good proliferation in 9 cases. Comparing biopsy or dissection of metastatic lesions, biopsy of primary lesions is much more reliable since prostate biopsy could be accomplished by transrectal or transperineal approach under local anaesthesia [14,25]. It could be easily performed in both treatment-naive patients and castration-resistant patients.

Currently, systematic therapy is still one of the most important treatment options for patients with PCa, especially advanced or locally advanced patients [26–28]. More clinically applicable drugs are being developed, followed by an increasing number of drug combinations. However, drug efficacy varies greatly between individuals due to the extensive intertumoral heterogeneity [29,30]. Therefore, drug sensitivity based on PDOs could provide precise information regarding the optimal treatment option or combination strategies. In the present study, our cultured PDOs were exposed to several common drugs for the treatment of advanced PCa, including second-generation hormonal therapy (Enzalutamide [31]), chemotherapy docetaxel [32] and poly (ADP-ribose) polymerase inhibitor (Olaparib [33]). Significant heterogeneity of treatment response was observed between the PDOs, suggesting that targeted biopsy-derived PDOs could serve as a preclinical model for individualized drug response to determine the optimal treatment. Targeted biopsy-derived PDOs could be used as a model to predict the treatment response of patients with locally advanced or advanced PCa to common drugs. These results would provide physicians with personalized information to make precise treatment strategies. Furthermore, our model has the potential to serve as a platform to uncover specific molecular alterations of PCa and explore new therapeutic targets [34].

Our studies still have some limitations. First, this study is a preliminary exploratory trial, proving the feasibility of PDOs based on targeted biopsy. More cases of targeted biopsy-derived PDOs and high-throughput drug screening results are needed to support its effectiveness. Second, it is difficult to carry out persistent and stable passage due to the limited proliferation of PDOs. Only two needles of biopsy tissue might be the key limiting factor for organoid culture. However, more side effects would happen if more biopsy cores are taken. Therefore, it seems to be beneficial to calculate the minimal tissue used for the development [35]. Technological innovation is still needed to optimize the culture of PDOs. Third, in order to improve the success rate of cultivation, we only selected tissues by targeted biopsy. Lacking verification of tissues by system biopsy is our defect. We will complete it when the technology improves in the future. Fourth, this study lacks clinical drug response of patients. It would be better to combine clinical validation to determine whether PDOs have the same drug response as patients in the clinic. It would be better to analyze the heterogeneity of treatment response by including the clinical features, such as Gleason scores, initial PSA, clinical staging, and gene alterations, in the multivariable analysis. However, the sample size of the current study is too limited to perform the multivariable analysis.

## 5. Conclusions

From our pilot study with a small sample size, we found that PCa PDOs based on targeted biopsy had similar histopathological and molecular features to the parental tumours. Our results also indicated that PDOs showed significant heterogeneity of treatment response, suggesting that PDOs could be developed as a platform to predict treatment response and help physicians to make optimal decisions.

## Supplementary Material

supplementary table materials.docx

## Data Availability

The data that support the findings of this study are available upon reasonable request.
