# Supplementary material for: Patient-derived organoids based on targeted biopsy of primary prostate cancer: development, identification, and drug screening
Source: Ann Med. 2025 Dec 16;57(1):2602324. doi: 10.1080/07853890.2025.2602324 (PMC12713212; doi:10.1080/07853890.2025.2602324)
Supplement: supplementary table materials.docx [file IANN_A_2602324_SM2340.docx]

**Supplementary table 1.** Basic information of the included patients

| ID | Age (years) | BMI (kg/m^2^) | PSA (ng/ml) | Prostate volume (ml) | Clinical T stage | PI-RADS score | Gleason score | Positive core |
| --- | --- | --- | --- | --- | --- | --- | --- | --- |
| 1 | 72 | 19.2 | 45.8 | 36.7 | T3a | 5 | 4+4 | 10 |
| 2 | 75 | 22.8 | 14 | 39.7 | T2c | 5 | 4+5 | 6 |
| 3 | 74 | 25.3 | 101 | 38.6 | T3b | 5 | 4+4 | 11 |
| 4 | 79 | 21.5 | 13 | 22.5 | T3a | 5 | 4+4 | 6 |
| 5 | 69 | 23.9 | 33.4 | 27.7 | T3a | 5 | 4+4 | 6 |
| 6 | 61 | 24.2 | 23.9 | 28.0 | T2c | 4 | - | - |
| 7 | 74 | 23 | 14.1 | 17.5 | T2c | 4 | 3+4 | 12 |
| 8 | 68 | 24.5 | 18.9 | 17.7 | T2c | 5 | 3+4 | 6 |
| 9 | 63 | 22.2 | 26.3 | 25.0 | T2c | 5 | 3+4 | 5 |
| 10 | 79 | 21.1 | 15.5 | 40.6 | T2c | 5 | 4+4 | 10 |
| 11 | 74 | 23.7 | 31.7 | 46.1 | T3b | 5 | 4+3 | 11 |
| 12 | 68 | 22.6 | 38.7 | 30 | T3a | 5 | 4+4 | 4 |
| 13 | 74 | 25.3 | 101 | 38.6 | T3b | 5 | 4+4 | 11 |

Abbreviation: BMI = Body mass index, PSA = Prostate specific antigen


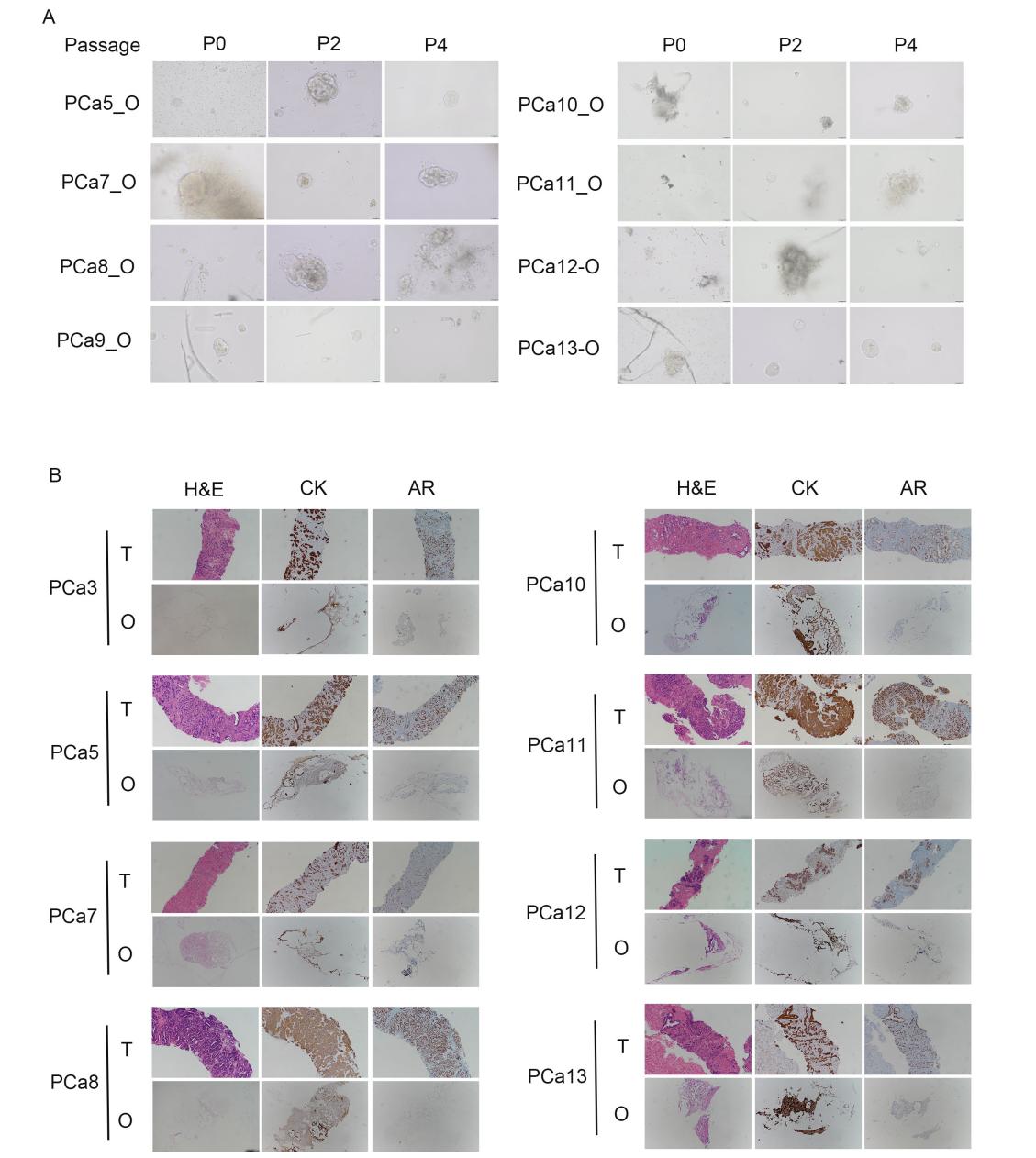


**Supplementary figure 1.** Histopathological characterization of prostate cancer PDOs (A) Staining smear of prostate cancer PDOs (X100). Scale bar, 200 mm. (B) H&E staining and immunohistochemistry staining of CK and AR on prostate cancer PDOs and corresponding parental tumors. Scale bar, 200 mm.
